# Dynamics of the soil microbial community associated with *Morchella* cultivation: diversity, assembly mechanism and yield prediction

**DOI:** 10.3389/fmicb.2024.1345231

**Published:** 2024-02-15

**Authors:** Yihong Yue, Haibo Hao, Qian Wang, Tingting Xiao, Yuchen Zhang, Qun Chen, Hui Chen, Jinjing Zhang

**Affiliations:** ^1^National Research Center for Edible Fungi Biotechnology and Engineering, Key Laboratory of Applied Mycological Resources and Utilization, Ministry of Agriculture, Shanghai Key Laboratory of Agricultural Genetics and Breeding, Institute of Edible Fungi, Shanghai Academy of Agricultural Sciences, Shanghai, China; ^2^State Key Laboratory of Genetic Engineering and Fudan Center for Genetic Diversity and Designing Agriculture, Institute of Plant Biology, School of Life Sciences, Fudan University, Shanghai, China; ^3^School of Biology Food and Environment, Hefei University, Hefei, China

**Keywords:** assembly processes, fungal richness, microbial community, morel cultivation, soil potassium, structural equation modeling (SEM)

## Abstract

**Introduction:**

The artificial cultivation of morels has been a global research focus owing to production variability. Understanding the microbial ecology in cultivated soil is essential to increase morel yield and alleviate pathogen harm.

**Methods:**

A total of nine Morchella cultivation experiments in four soil field types, forest, paddy, greenhouse, and orchard in Shanghai city were performed to determine the potential ecological relationship between Morchella growth and soil microbial ecology.

**Results:**

Generally, significant variation was observed in the soil microbial diversity and composition between the different experimental field types. The niche width analysis indicated that the bacterial habitat niche breadth was significantly greater than the fungal community width, which was further confirmed by a null model that revealed that homogeneous selection could explain 46.26 and 53.64% of the variance in the bacterial and fungal assemblies, respectively. Moreover, the neutral community model revealed that stochastic processes dominate the bacterial community in forests and paddies and both the bacterial and fungal communities in orchard crops, whereas deterministic processes mostly govern the fungal community in forests and paddies and both the bacterial and the fungal communities in greenhouses. Furthermore, co-occurrence patterns were constructed, and the results demonstrated that the dynamics of the soil microbial community are related to fluctuations in soil physicochemical characteristics, especially soil potassium. Importantly, structural equation modeling further demonstrated that the experimental soil type significantly affects the potassium content of the soil, which can directly or indirectly promote Morchella yield by inhibiting soil fungal richness.

**Discussion:**

This was the first study to predict morel yield through soil potassium fertilizer and soil fungal community richness, which provides new insights into deciphering the importance of microbial ecology in morel agroecosystems.

## 1 Introduction

Morel belongs to an ascomycetous mushroom genus and is widely distributed in North America, Europe and Asia (Benucci et al., 2019), especially in the temperate regions of China (Sambyal and Singh, 2021; Yu Y. et al., 2022). As a valuable edible fungus with important economic and scientific value, morel has been successfully domesticated and cultivated on a large scale in ordinary agricultural soils worldwide in recent years (Tan et al., 2019; Xu et al., 2022). However, the cultivation process of morel is unstable, and its planting fields have suffered serious losses in fruiting body yield, even with no harvest, resulting in catastrophic economic losses and seriously hindering the development of the morel agroindustry. Previous studies have shown that the variability in *Morchella* yield is due to the vitality of cultivated strains, field management models (Zhang C. et al., 2023), soil-borne pathogens (Guo et al., 2016; He et al., 2017; Lan et al., 2020), soil physicochemical characteristics (Zhang et al., 2019), volatile organic compounds (VOCs) (Yu F. et al., 2022), genetic instability (Chai et al., 2017), and microbial community evenness (Tan et al., 2021a,b). Differences in *Morchella* growth were observed between the different habitats. The yield of *Morchella* cultivated in greenhouse soil significantly increased (Liu et al., 2018). Intercropping mode of fruit*-Morchella* improved soil structure and fertility (Song et al., 2021), and the total amino acid content of cultivated *Morchella* was greater than that of field-planted *Morchella* (Wei et al., 2020). A high *Morchella* density was also observed in plants in mechanically injured or burned conifer forest environments (Goldway et al., 2000; Kuo et al., 2012; Li et al., 2017). To date, the impact of soil type on fruiting body formation in *Morchella* has been underestimated, and it is necessary to determine the suitable soil type for *Morchella* cultivation.

Notably, a recent review reported that the microecology of cultivated soil has a greater impact on *Morchella* than the physicochemical characteristics of the soil (Xu et al., 2022). Indeed, soil microorganisms affect *Morchella* growth by decomposing and accumulating nutrients (Benucci et al., 2019), while *Morchella* fruiting is significantly positively correlated with soil microbial community diversity and evenness (Tan et al., 2021a,b). The soil microbiota can serve as a biomarker for specific growth stages during mushroom cultivation, which is beneficial for mushroom development and has potential for use as an inoculant (Singh et al., 2022). *Bradyrhizobium* and *Sphingomonas* have strong lignocellulose degradation abilities, which help to elongate mycelia and metabolize nutrients during the mycelial stage (Gohar et al., 2022). *Pseudomonas* species can promote mycelial growth, increase yield, and directly interact with mushrooms during the primordium stage to induce primordium formation (Cho et al., 2003; Kim et al., 2008; Zarenejad et al., 2012). Members of *Burkholderiaceae* can promote ascocarp growth during the fruiting stage (Zhang Y. et al., 2023). However, the soil substrate in cultivated mushrooms transmits potential pathogenic fungal taxa, including *Aspergillus, Fusarium, Penicillium*, and *Trichoderma*, leading to a decrease in yield and quality (Lo Cantore and Iacobellis, 2014). Some fungal species inhibit *Morchella* mycelial growth by secreting antagonistic exudates (Wang et al., 2020). The main reason for the decline in the yield of *Morchella* under continuous cropping cultivation is also related to the proliferation of pathogenic fungi, which leads to a decrease in soil fungal diversity and ecological niches (Liu et al., 2022). Importantly, the pathogen load may increase from the primordium stage to the fruiting body production stage in *Morchella* (Zhang C. et al., 2023). Therefore, exploring the potential relationship between microbial ecology and *Morchella* yield in soil agroecosystems is essential for the development of the *Morchella* agroindustry.

Microbial ecology is critical to agricultural ecosystems for soil mushroom cultivation, especially for the unstable fruit yield of *Morchella*. In this study, nine *Morchella* cultivation experiments were performed for four soil field types, forest, paddy, greenhouse, and orchard, in Shanghai city to determine the potential ecological characteristics of the bacterial and fungal communities during fructification. The aims were to (1) compare the diversity and composition of bacterial and fungal communities between different experimental field types, (2) dissect the co-occurrence patterns and community assembly mechanism, and (3) assess the potential ability of bacterial and fungal communities to predict *Morchella* yield.

## 2 Materials and methods

### 2.1 Study area selection and soil sample collection

A total of nine experimental fields of *Morchella* cultivation of four different types were selected in the suburban area around Shanghai city; these included three fields from forests, two fields from paddies, three fields from greenhouses, and one from an orchard. For each field, the five-point sampling method was used to collect soil samples in March 2022 with *Morchella* fruiting, after which five parallel samples were taken at soil depths of 0–10 cm (topsoil) and 10–20 cm (subsoil). Subsequently, each soil sample was divided into two portions: One was placed at 4°C for soil physiochemical measurements, and the other was stored at−80°C for molecular experiments. Moreover, the number of ascocarps per square meter was recorded as the yield level of each experimental field.

### 2.2 Soil physiochemical analysis

The alkaline hydrolysable nitrogen (AN), soil organic matter (SOM), available potassium (AK), total phosphorus (TP), available phosphorus (AAP), organic carbon (OC), cellulose, hemicellulose, sugar, glucose, fructose, saccharose, and starch were measured according to the corresponding assay kits (Comin Biotechnology, Suzhou, China, http://www.cominbio.com), including soil alkaline hydrolysable nitrogen content assay kit (SSN-2-Y), soil organic matter content assay kit (SOM-1-T), soil available potassium content assay kit (SSJ-2-G), soil total phosphorus content assay kit (SQL-1-G), soil available phosphorus kit (NKSXL-1-G), soil organic carbon content assay kit (SOC-1-T), cellulose content assay kit (CLL-1-Y), hemicellulose content assay kit (BXW-1-G), total sugar content assay kit (ZT-1-Y), glucose content assay kit (PT-1-Y), fructose content assay kit (GT-1-Y), saccharose content assay kit (ZHT-1-Y), and starch content assay kit (DF-1-Y), respectively. The soil pH, total nitrogen (TN), and total potassium (TK) were measured using pH meters, Kjeldahl nitrogen determination, and flame photometry, respectively.

### 2.3 Soil DNA extraction and high-throughput sequencing

Using the E.Z.N.A@Soil DNA Kit (Omega Biotek, Norcross, GA, U.S.), the soil genomic DNA was extracted from each sample. The primers 341F (5′ – CCTAYGGGRBGCASCAG - 3′)/806R (5′ – GGACTACNNGGGTATCTAAT - 3′) and ITS1F (5′ – CTTGGTCATTTAGAGGAAGTAA - 3′)/ITS2R (5′ – GCTGCGTTCTTCATCGATGC - 3′) were used to amplify the V3-V4 region of the bacterial 16S rDNA and the ITS1 region of the internal transcribed spacer (ITS) region of the fungus, respectively. PCR was performed in 20 μL reactions with 4 μL of 5 × FastPfu Buffer, 5 μM forward and reverse primers, 2.5 mM dNTPs, 0.4 μL of FastPfu Polymerase, and 10 ng of template DNA. The purified and quantified PCR products were obtained using an AxyPrep DNA Gel Extraction Kit (Axygen Biosciences, Union City, CA, U.S.) and Qubit^®^3.0 (Thermo Fisher Scientific, Waltham, MA, USA), respectively. Then, the amplicon libraries were sequenced on an Illumina MiSeq PE250 platform (Biozeron Biological Technology Co., Ltd., Shanghai, China) using standard protocols (http://www.illumina.com/).

### 2.4 Data analysis

After sequencing, the obtained raw fastq data were analyzed via the QIIME2 pipeline (http://qiime.org/scripts/assign_taxonomy.html). The primer and index barcode sequences were first removed and merged into a paired-end sequence. Then, through quality control, denoising and chimerism removal, the optimized bacterial and fungal ASVs were clustered against the Silva database (Release 138) and UNITE v7.2 (Full UNITE + INSD datasets) according to a similarity threshold of 97%. Sequencing scales were normalized to the lowest number of sequences among all samples.

### 2.5 Statistical analyses

With Mothur software (version 1.30.2, https://www.mothur.org/wiki/Download_mothur), the taxonomic richness, evenness, and diversity of alpha diversity were estimated using the Chao1, Pielou_J, Shannon and PD_faith indices (Schloss et al., 2009). Based on the Bray–Curtis distance, beta diversity was calculated through principal coordinates analysis (PCoA) to determine the similarities or differences in microbial communities between different samples using the R “vegan” package. PCoA is a nonconstrained data dimensionality reduction analysis method in which potential principal components that affect the differences in sample community composition are identified through dimensionality reduction.

Linear regression is a statistical analysis method that uses regression analysis in mathematical statistics to determine the relationship between one or more independent and dependent variables, where *R*^2^ is the coefficient of determination, representing the proportion of variance explained by the regression line. The Mantel test was used to test the correlation between community distance matrices (UniFrac distance matrices) and the environmental variable distance matrix with the R corrplot package. A co-occurrence network was constructed to calculate the correlation between species using Sparse Correlations for Compositional Data (SparCC), which was visualized in Gephi (v0.9.2) and Cytoscape (v3.6.1) (Barberán et al., 2012). When the correlation coefficient reached a certain threshold with a SparCC-r value ≥ 0.8 and a statistically significant *p* value < 0.05, there was a connection between species (Junker and Schreiber, 2008). Furthermore, through the R packet graph, the topological properties were used to calculate the node degree distribution, average degree, diameter, average path length, density, clustering coefficient, modularity, etc., to further obtain intragroup or intergroup related information of the species (Csardi, 2013).

The neutral community model (NCM) was mainly used to quantify the ecological importance of stochastic processes with R version 3.6.1 (Sloan et al., 2006). *R*^2^ represents the overall goodness of fit of the model at 95% confidence intervals, and the higher the value is, the greater the impact of stochastic processes on the microbial community. Nm is the product of metacommunity size and migration rate and determines the correlation between occurrence frequency and relative abundance (Burns et al., 2016). Structural equation modeling (SEM) is a method to establish, estimate, and test causal relationship models by AMOS 21.0.0 using the maximum likelihood estimation method. The model included observable explicit variables as well as potential variables that could not be directly observed; these variables could be used to identify the impact of individual indicators on the population and the interrelationships between individual indicators. The fit of the suitable model was judged by the non-significant chi-square test (*p* > 0.05), high goodness-of-fit index (GFI > 0.90), and low root mean square errors of approximation (RMSEA < 0.05). Random forest (RF) regression analysis is an integrated learning technique that can be used to analyze large-scale datasets effectively. It is a powerful nonlinear regression model that fits characteristic variables with response variables.

## 3 Results

### 3.1 Physicochemical variables of soil in different types of experimental fields

**Figure 1:**
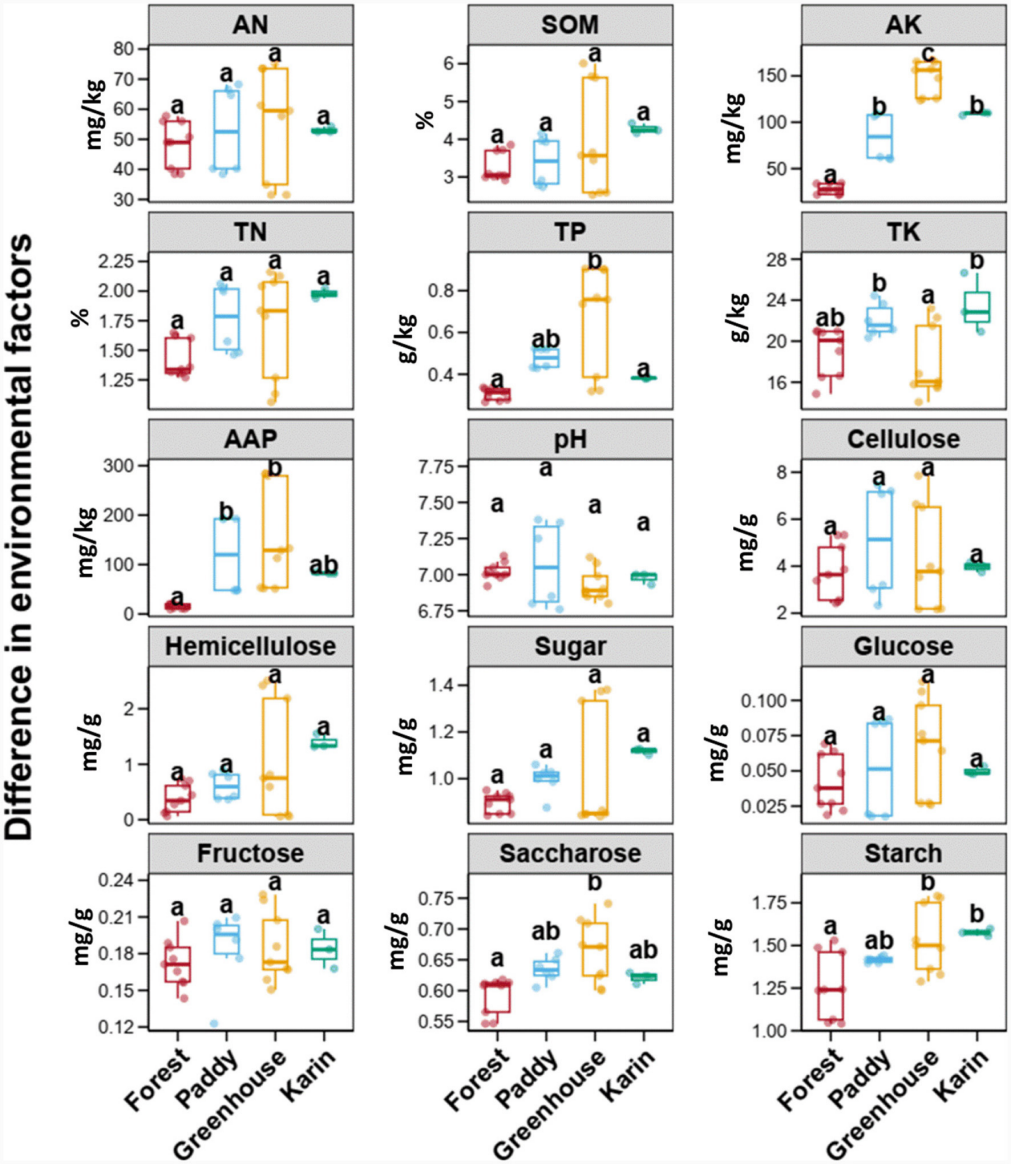
Boxplots of the soil physicochemical variables among different types of experimental fields. Error bars represent the standard deviation of the mean (*n* ≥ 5). Numbers in a rank with different letters indicate a significant difference (Tukey's HSD test, *p* < 0.05). AN, available nitrogen; SOM, soil organic matter; AK, available potassium; TN, total nitrogen; TP, total phosphorus; TK, total potassium; AAP, alkaline available phosphorus.

To understand the soil environment in which *Morchella* was cultivated, the variations in the soil physicochemical characteristics in the different experimental field types were compared through Tukey's HSD test (Figure 1). There was no significant difference in soil AN, SOM, TN, pH, cellulose, hemicellulose, sugar, glucose, and fructose among the four fields (*p* > 0.05). The contents of AK, TP, APP, saccharose and starch were the highest in the greenhouse, followed by those in the paddy and orchard fields, while those in the forest were the lowest (*p* < 0.05). The TK in paddy and orchard fields had relatively greater values than TK in forests and greenhouses (*p* < 0.05).

### 3.2 Microbial community diversity between different soil depths and field types

**Figure 2:**
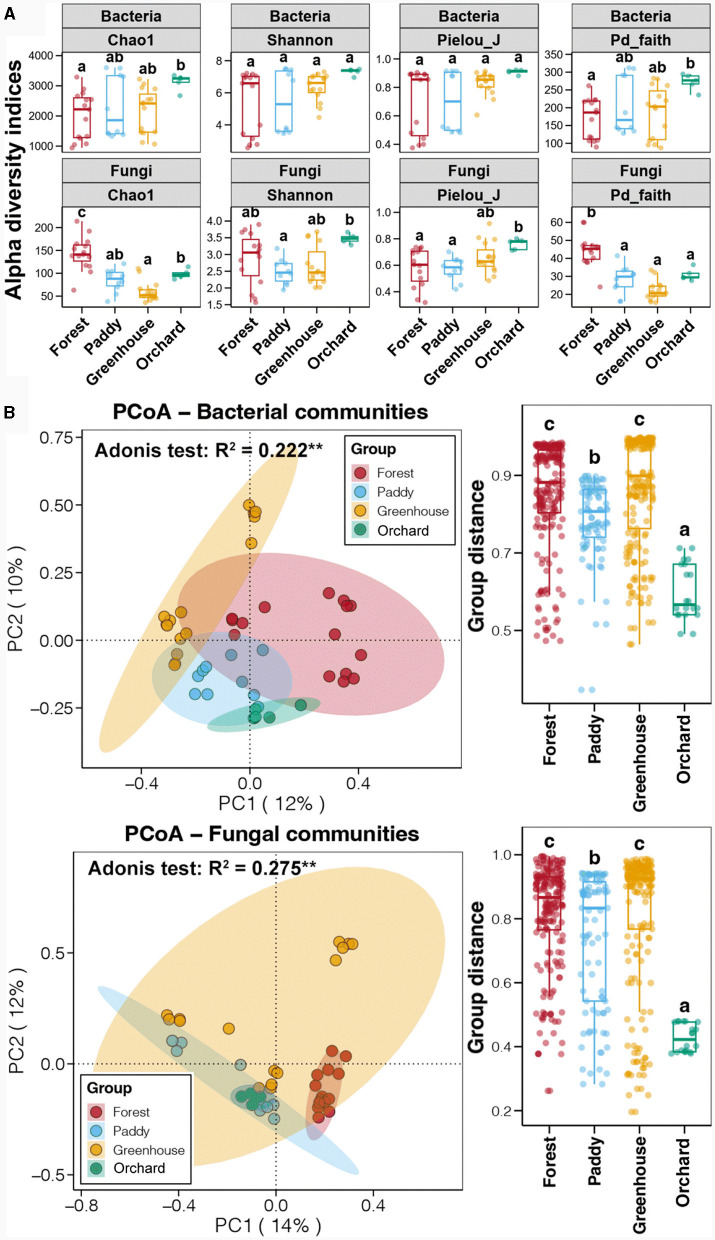
The alpha diversity **(A)** and principal coordinates analysis (PCoA) **(B)** of the bacterial and fungal communities among the different experimental field types. Error bars represent the standard deviation of the mean (*n* ≥ 5). Numbers in a rank with different letters indicate a significant difference (Tukey's HSD test and Adonis test, *p* < 0.05).

Ninety soil samples from nine experiments on four soil field types, namely, forest (three), paddies (two), greenhouse (three), and orchard (one), were collected at soil depths of 0–10 cm (topsoil) and 10–20 cm (subsoil) to determine the bacterial and fungal communities in the *Morchella* cultivated soil. Sequencing of the bacterial and fungal communities was conducted for 45 topsoil samples, with 3,722,811 and 5,074,587 effective sequences obtained, respectively (Supplementary Table 1). The soil bacterial Chao1 and Pd_faith indices were the highest in the field, followed by those in the greenhouse and paddy fields, with the lowest values occurring in the forests (p < 0.05) (Figure 2A). There was no significant difference in the Shannon or Pielou_J indices of the soil bacteria between the different experimental field types (*p* > 0.05). Unlike the alpha diversity of the soil bacteria, the soil fungi had the highest Chao1 and Pd_faith indices in the forest, followed by those in the orchard and paddy fields, with the lowest values occurring in the greenhouse (*p* < 0.05). The Shannon and Pielou_J indices of soil fungi in the orchard fields were the highest, followed by those in the forests and greenhouses, and they were the lowest in the paddy fields (*p* < 0.05). These results reflected the variation in alpha diversity among the different Morchella cultivated fields. The alpha diversity analysis showed that when considering only the soil depth or field type as a single factor, no significant differences were observed in the soil bacterial diversity (*p* > 0.05) (Supplementary Figure 1A). In particular, the analysis of the bacterial alpha diversity in the soils of the different experimental types at the same depth revealed that the Chao1 and Pd_faith indices in the topsoil were significantly different (*p* < 0.05), and no significant differences were detected in the subsoil (*p* > 0.05).

Furthermore, principal coordinate analysis (PCoA) was used to evaluate the effects of soil depth and field type on the soil bacterial composition based on the Bray–Curtis distance (Supplementary Figure 2A). The soil bacterial communities at different soil depths were similar, while at different experimental soil types, they showed significant differences. Furthermore, the results of the diversity analysis of the soil fungal communities were similar to those of the bacterial communities (Supplementary Figures 1B, 2B). Above all, the soil depth did not significantly impact the bacterial or fungal diversity, whereas the experimental field type was significantly correlated with the soil bacterial and fungal communities. This difference might have been related to the fact that the mycelial networks of *Morchella* were basically between the 0–10 cm soil depths. Therefore, it was determined that the microbial community in the subsoil was not associated with *Morchella* cultivation, and subsequent analysis focused on the topsoil samples. A PCoA based on the Bray–Curtis distance showed that the soil bacterial communities in the different experimental field types were clustered separately (Figure 2B). The Adonis test further revealed that the field type significantly impacted the soil bacterial community, which could have explained 22.2% of the soil bacterial community variation. A comparison of the Bray–Curtis distances between samples from the same field type showed that the difference in the soil bacterial community between the forests and greenhouses was the greatest, followed by that in the paddy field, with the lowest difference occurring in the orchard field (*p* < 0.05). Similarly, the soil fungal communities were also divided into four experimental field types. The Adonis test further revealed that the field type significantly impacted the soil fungal community and explained 27.5% of the soil fungal community changes. A comparison of the Bray–Curtis distances between samples of the same field type showed that the difference in the fungal community was greatest in the forest and greenhouse soils, followed by that in the paddy soil, and that in the orchard soil was the lowest (*p* < 0.05), which was consistent with the dynamic changes in the bacterial community. Thus, there were also significant differences in microbial diversity between soils cultivated with Morchella in the same experimental field type.

The effective annotation of the bacterial ASVs was 100% at the phylum level, approximately 70% at the family level, and only 43.32 and 3.35% at the genus and species levels, respectively, with a total of 51 phyla, 140 classes, 309 orders, 421 families, 1,105 genera, and 888 species annotated (Supplementary Figure 3A). Moreover, the effective annotation of fungal ASVs at the phylum level was 92.69%, which could also reach approximately 70% at the family level, and 57.64 and 73.42% at the genus and species levels, respectively; additionally, eight phyla, 18 classes, 47 orders, 95 families, 119 genera, and 175 species were annotated. The relatively higher fungal species annotation was due to the existence of unknown specific taxonomic information in the database, which was not a true annotation value. In addition, the coefficient and accumulation curves of the bacterial and fungal communities tended to form straight lines, indicating that sequencing depth could effectively represent community information, which was sufficient to represent the research system (Supplementary Figures 3B, C).

### 3.3 Microbial composition and indicator taxa among different experimental field types

**Figure 3:**
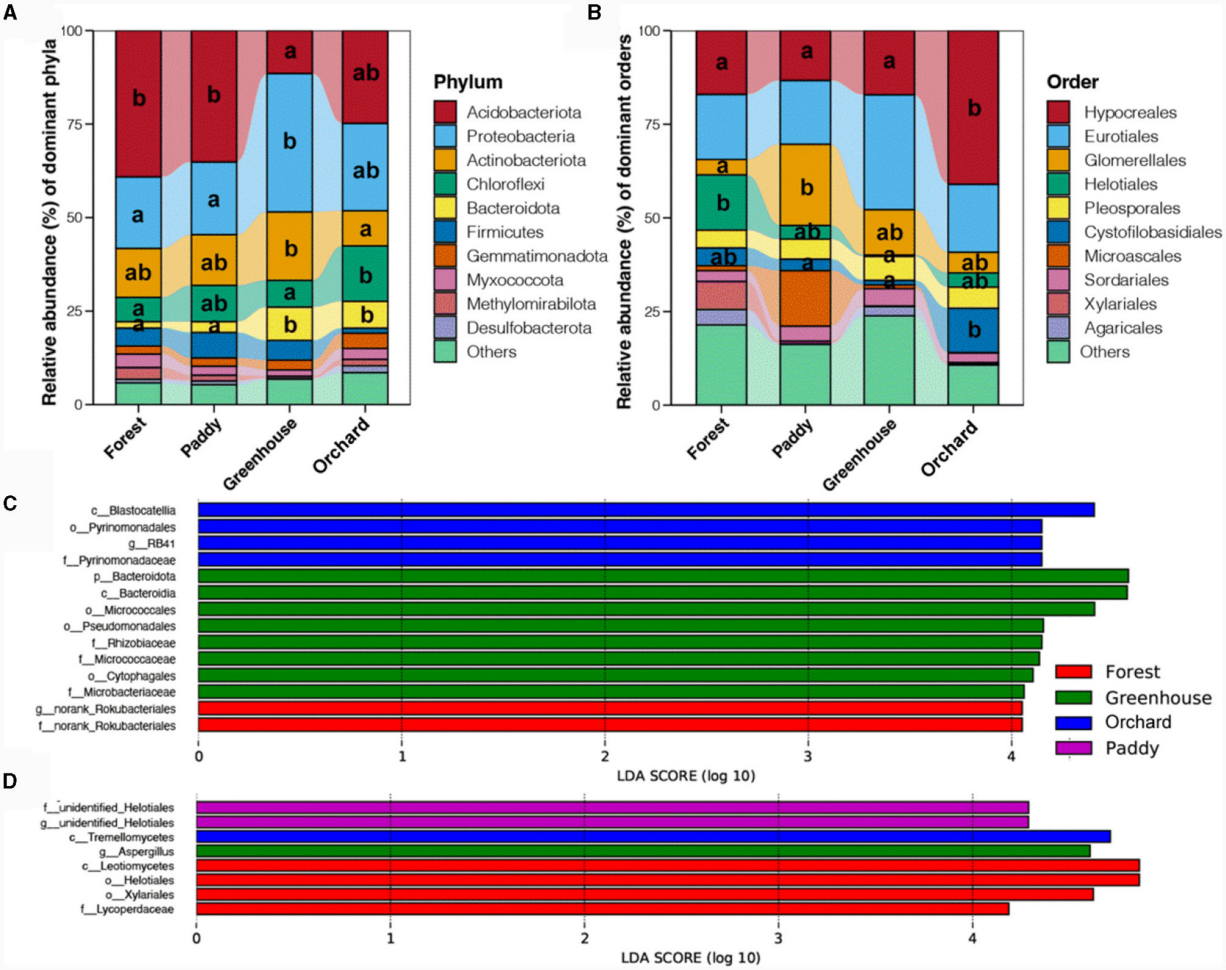
Dominant bacterial phyla **(A)** and fungal orders **(B)** together with indicator bacterial **(C)** and fungal **(D)** taxa in different experimental field types. Different lowercase letters indicate a significant difference between different cultivation experiments in four soil field types (*p* < 0.5).

At the phylum level, the soil bacterial community was mainly composed of *Acidobacteriota* (27.66%), followed by *Proteobacteria* (24.71%), *Actinobacteriota* (13.58%), *Chloroflexi* (9.53%), and *Bacteroidota* (5.19%) (Figure 3A). The relative abundance of *Acidobacteriota* was relatively greater in forests and paddy fields, followed by orchard and greenhouse (*p* < 0.05), which was opposite to the distribution of *Proteobacteria* (*p* < 0.05). *Actinobacteriota* had the highest proportion in greenhouses, followed by forests and paddies, and the lowest proportion was found in orchard (*p* < 0.05). The abundance of *Chloroflexi* was highest in orchard, followed by paddy, and lowest in forests and greenhouses (*p* < 0.05). The abundance of *Bacteroidota* was significantly greater in the greenhouse and orchard crops than in the forests and paddy fields (*p* < 0.05). Among the different fungal community compositions, *Ascomycota* and *Basidiomycota* were the most abundant phyla in the soil (Supplementary Figure 4). *Basidiomycota* had the highest proportion in the forests, followed by orchard and greenhouse, and it had the lowest proportion in the paddy field (*p* < 0.05), while the proportion of Ascomycota was quite similar (*p* > 0.05). At the order level, *Hypocreales* (22.13%) and *Eurotiales* (20.80%) were the predominant fungi, followed by *Glomerellales* (11.70%), *Helotiales* (10.85%), *Pleosporales* (5.63%), and *Cystofilobasidiales* (5.55%) (Figure 3B). *Hypocreales* and *Cystofilobasidiales* had higher abundances in orchard, while the abundance of *Helotiales* was greater in the forests.

Linear discriminant analysis effect size (LefSe) was used to identify bacterial and fungal taxa that were predictive of the different *Morchella* cultivated fields. For the soil bacterial taxa (Figure 3C), g_norank_*Rokubacteriales* and f_norank_*Rokubacteriales*, which belong to *Methylomirabilota* were significantly enriched in the forest, while a variety of bacteria were significantly enriched in the greenhouse, including f_*Rhizobiaceae*, which belongs to *Alphaproteobacteria*; f_*Micrococcaceae*, which belongs to *Actinobacteriota*; o_*Pseudomonadales*, which belongs to *Gammaproteobacteria*; and o_*Cytophagales*, which belongs to *Bacteroidota*. c_*Blastocatellia*, together with o_*Pyrinomonadales* and g_RB41, which belong to *Acidobacteria*, were significantly enriched in orchard. For the soil fungal taxa (Figure 3D), o_*Helotiales*, o_*Xylariales*, and f_*Lycoperdaceae*, which belong to *Agaricales*, were significantly enriched in the forest, while g_*Aspergillus*, which belongs to *Eurotiales*, was significantly enriched in the greenhouse. c_*Tremellomycetes* was significantly enriched in the orchard soil, and two unidentified fungi belonging to *Helotiales* were significantly enriched in the paddy soil. These results indicated that most of the dominant bacterial phyla and fungal orders in the microbial community matched the results determined by LefSe and could serve as indicators of bacteria and fungi that were mostly related to the microbial community in different experimental field types of *Morchella* cultivation.

### 3.4 Relationships between physicochemical variables and the soil microbial community

**Figure 4:**
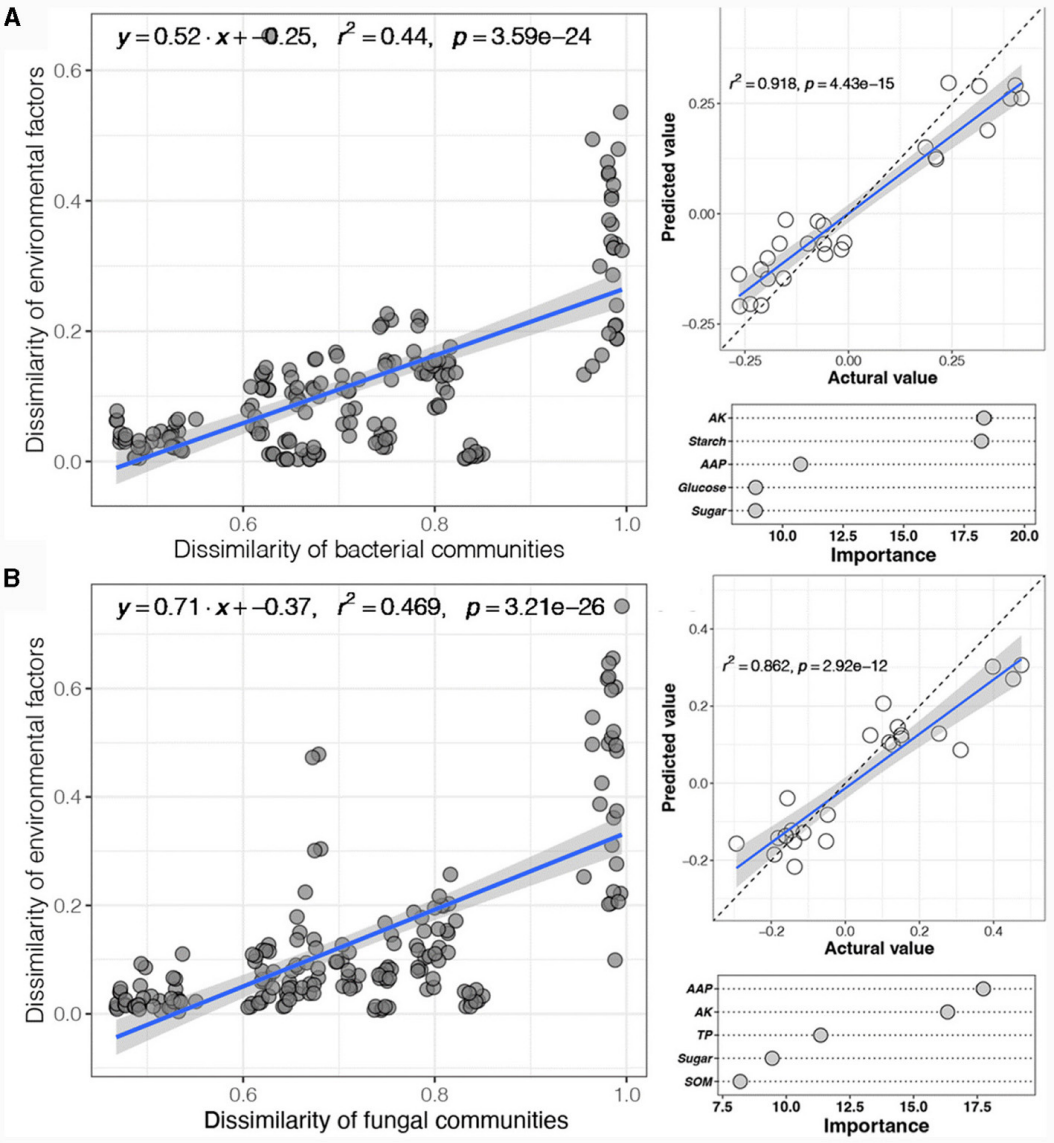
Correlations between physicochemical variables and soil microbial community according to linear regression **(A)** and random forest analysis **(B)**.

Subsequently, the correlation between the physicochemical variables and the soil microbial community was evaluated through distance-dependent similarity (Figure 4). The results showed that both the bacterial and fungal communities exhibited significant environmental distance-dependent similarity (*p* < 0.05) (Figures 4A, B). The environmental distance-dependent similarity and regression curve slope of the fungal community were slightly greater than those of the bacterial community, indicating that the correlation between physicochemical variables and the soil fungal community was greater than that between the soil fungal community and the bacterial community. Furthermore, random forest analysis was used to infer the major significant predictors of physicochemical variables influencing the microbial community. The results revealed that physicochemical variables had great predictive ability for changes in soil bacterial and fungal communities. Among them, the physicochemical variables significantly associated with the soil bacterial community were AK, starch, AAP, glucose, and sugar (Figure 4C), while those associated with the soil fungal community were AAP, AK, TP, sugar, and SOM (Figure 4D).

**Figure 5:**
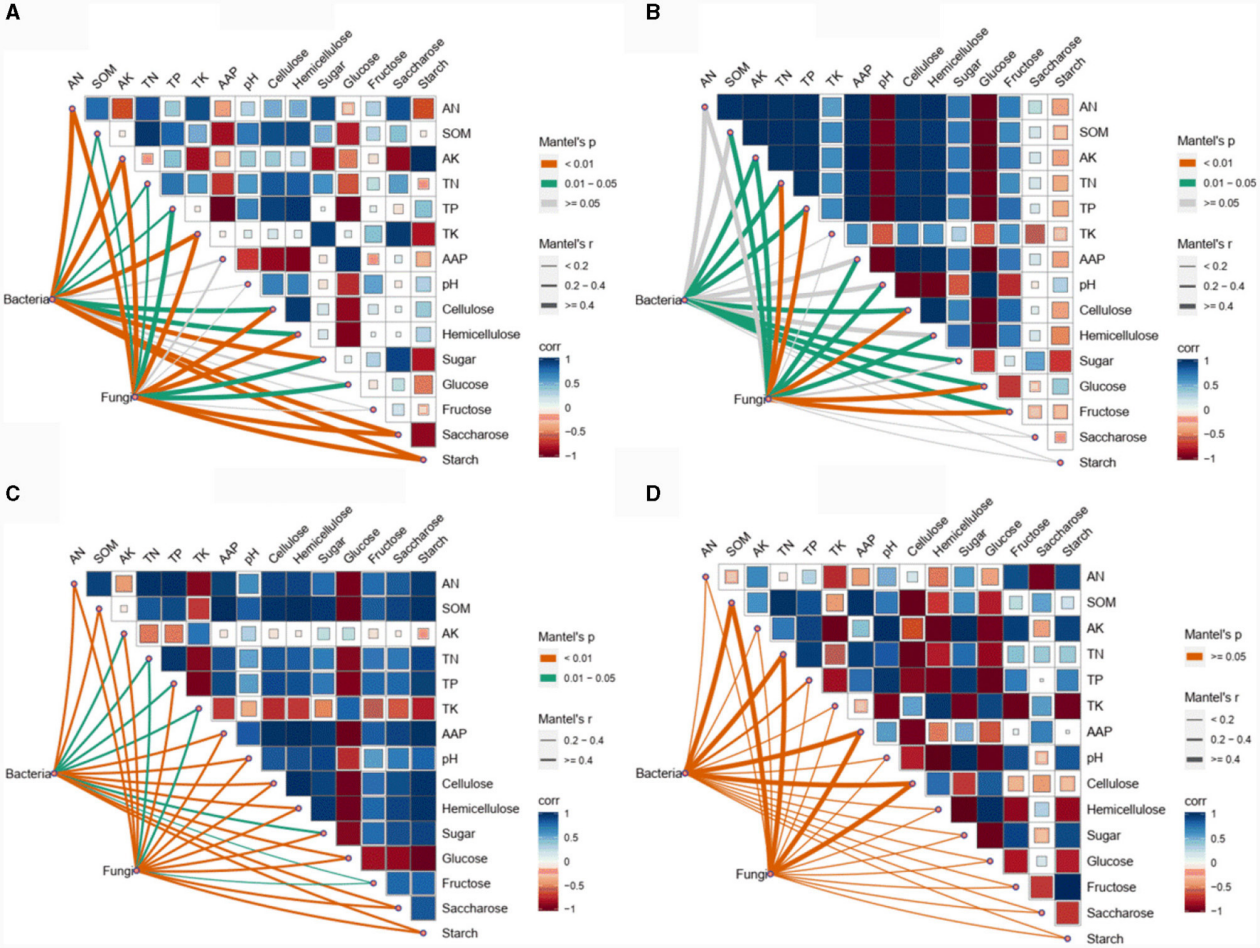
Correlations of the soil physicochemical variables with bacterial and fungal communities in forest **(A)**, paddy **(B)**, greenhouse **(C)**, and orchard **(D)** plots.

The Mantel test was carried out to examine the physicochemical variables significantly correlated with the soil bacterial and fungal communities among the different experimental field types (Figure 5). In the forests, AK, starch, AN, sugar, saccharose, and TK were the 5 physicochemical variables (mantel-r ≥ 0.4, *p* < 0.01) influencing the bacterial community, while starch, AK, AN, and TK (Mantel-r ≥ 0.4, *p* < 0.01) were significantly correlated with the fungal community (Figure 5A). In the paddy field, the bacterial community showed significantly negative correlations with cellulose, TP, glucose, AK, TN, fructose, and sugar (Mantel-r ≥ 0.4, *p* < 0.05), while the fungal community was significantly positively correlated with cellulose, TN, TP, glucose and fructose (Mantel-r ≥ 0.4, *p* < 0.01), and negatively correlated with AAP, AK, SOM, pH and hemicellulose (Mantel-r ≥ 0.4, *p* < 0.05) (Figure 5B). Notably, all the physicochemical variables were significantly correlated with the bacterial and fungal communities in the greenhouse (Mantel-r ≥ 0.4 or Mantel-r 0.2-0.4, *p* < 0.01 or *p* < 0.05) (Figure 5C). In contrast, no significant correlations were observed between the bacterial or fungal communities and any of the other physicochemical variables in orchard (*p* > 0.05) (Figure 5D). These findings indicated that soil physicochemical variables were unlikely to be decisive factors in determining the dynamics of microbial composition in different experimental field types of *Morchella* cultivation.

### 3.5 Relative importance of deterministic and stochastic processes in the assembly mechanisms of bacterial and fungal communities

**Figure 6:**
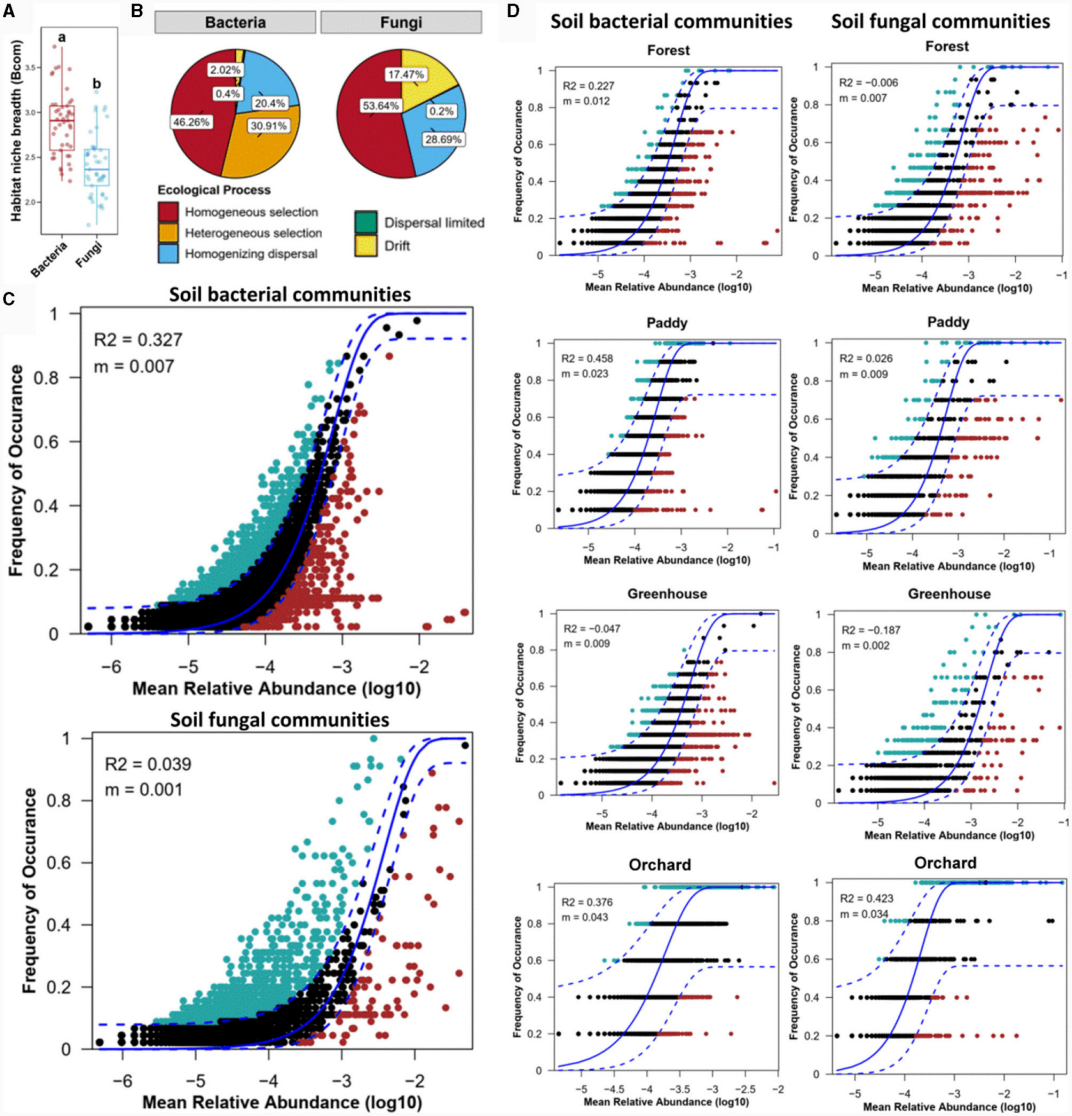
Ecological processes shaping the bacterial and fungal communities in different experimental field types through the niche width **(A)**, null model **(B)**, and neutral community model (NCM) **(C, D)**.

To reflect the adaptation of species to the environment, the niche width of the microorganisms was calculated (Figure 6A). Niche width analysis revealed that the habitat niche breadth of bacteria was greater than that of fungi, indicating that the fungal community in the cultivated soil of *Morchella* was more sensitive to the bacterial community. This finding was consistent with the results showing environmental distance-dependent similarity (Figures 4A, B). Furthermore, the null model was used to analyze the assembly mechanism of the bacterial and fungal communities (Figure 6B). The ecological process data demonstrated that homogeneous and heterogeneous selection of deterministic processes (77.17%) were the dominant drivers shaping the soil bacterial community, while homogeneous selection of deterministic processes (53.64%) affected the soil fungal community. Despite the relatively lower contribution of deterministic processes to fungal community assembly, homogeneous selection in the fungal community (53.64%) was significantly greater than that in the bacterial community (46.26%) among the different experimental field types. These results indicated that deterministic processes contributed to both bacterial and fungal community assembly.

Furthermore, the neutral community model (NCM) elucidated the relationship between the occurrence frequency and relative abundance of ASVs (Figure 6C). Stochastic processes were the more dominant factors affecting the soil bacterial community than the fungal community. These findings revealed that deterministic and stochastic processes together shaped the bacterial community, while the fungal community was governed mainly by deterministic processes among the different experimental field types. Notably, the assembly mechanisms of the microbial communities in the *Morchella* cultivation soils of the different experimental field types were not similar (Figure 6D). For the bacterial community, stochastic processes in the paddy field (*R*^2^ = 0.458) were more common than those in orchard (*R*^2^ = 0.376) and forest (*R*^2^ = 0.227), while stochastic processes did not affect the bacterial community assembly in the greenhouse. For the fungal community, only orchard was significantly shaped by stochastic processes, with an *R*^2^ = 0.423. Above all, stochastic processes dominated the bacterial community in forests and paddies and both bacterial and fungal communities in orchard crops, whereas deterministic processes mostly governed the fungal community in forests and paddies and both bacterial and fungal communities in greenhouses. These findings were consistent with the results of the Mantel test (Figure 5).

### 3.6 Co-occurrence patterns of bacterial and fungal communities

**Figure 7:**
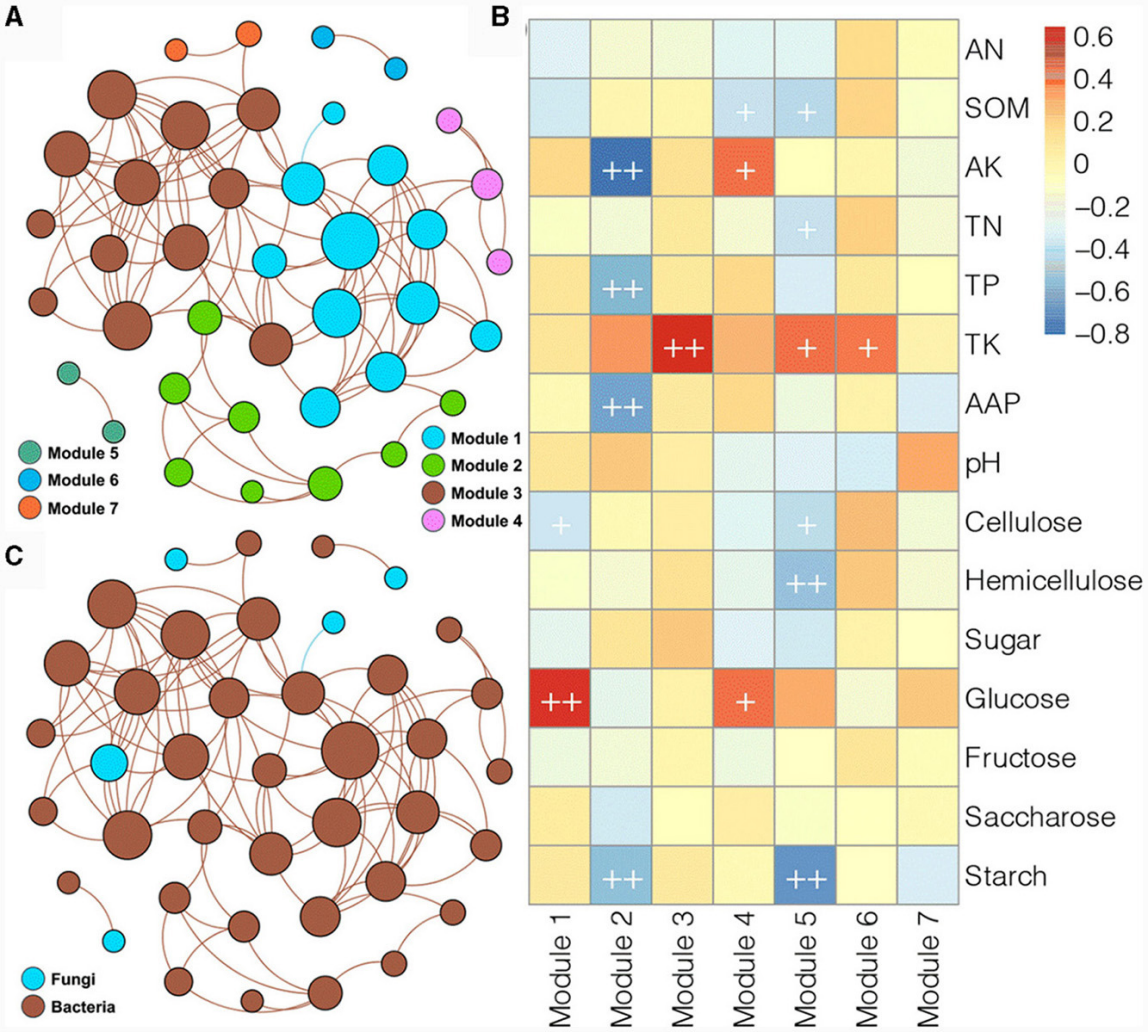
Co-occurrence networks of bacterial and fungal ASVs colored according to module **(A)** and taxonomy **(B)** and the associations between modules and physicochemical variables **(C)**.

Due to the limited number of fungal species and significant differences between samples, the soil bacterial and fungal communities from the different experimental field types of *Morchella* cultivation were combined to construct a co-occurrence network (Figure 7). The obtained network consisted of 40 nodes and 105 edges, with an average node degree of 5.25 and a modularity index of 0.503, clustering into seven major modules (Figure 7A). Among them, module I, module II, and module III had more species than the other four modules. Most of the associations in the network were associated with bacterial species, and there were only five fungal nodes; four fungal nodes were related to only one bacterial node, and only one fungal node was related to multiple bacterial nodes (Figure 7B). Moreover, most of the associations in the network were positive, and only a negative correlation between one bacterial and one fungal node was detected with a blue line. Spearman correlation analysis was further performed to evaluate the correlation between the modules and soil physicochemical variables (Figure 7C). Module I was positively correlated with glucose and negatively correlated with cellulose. Module II was negatively correlated with AK, TP, AAP, and starch. Module III was positively related to TK. Module IV was positively correlated with AK and glucose and negatively related to SOM. Module V was positively related to TK but negatively correlated with SOM, TN, cellulose, hemicellulose, and starch. Module VI was positively correlated with TK. These results implied that the dynamics of the soil microbial community were related to fluctuations in soil physicochemical characteristics in the different experimental field types of *Morchella* cultivation.

### 3.7 Relationships between the soil microbial community and *Morchella* yield

**Figure 8:**
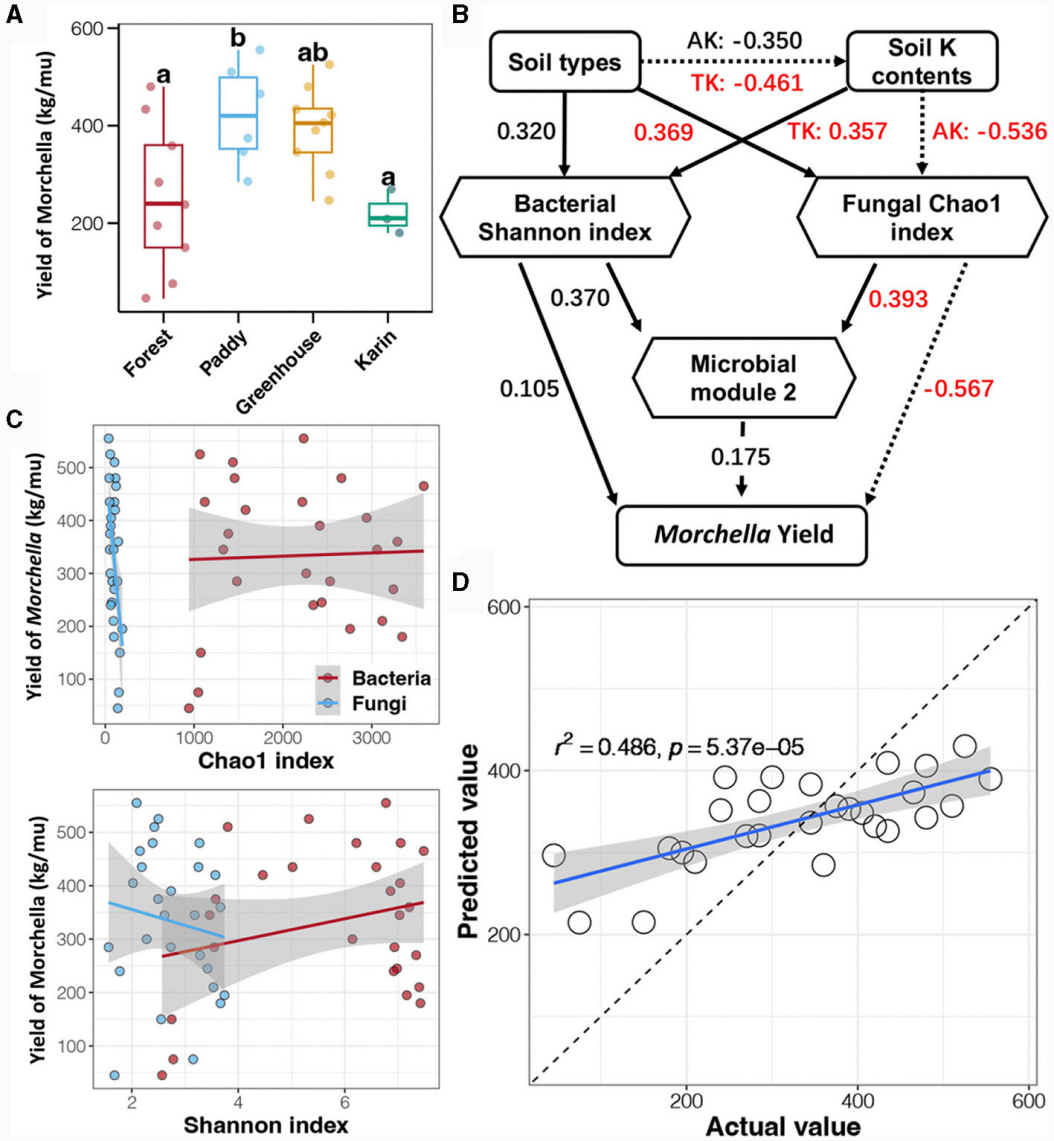
*Morchella* yield **(A)** and its relationship with the soil microbial community based on linear regression analysis **(B)**, structural equation modeling (SEM) **(C)**, and random forest (RF) regression analysis **(D)**. Different lowercase letters indicate a significant difference between different cultivation experiments in four soil field types (*p* < 0.5).

To explore the extent to which the microbial community impacted the *Morchella* yield, the relationship between the soil microbial community and the *Morchella* yield was evaluated (Figure 8). According to the Tukey's HSD test, the *Morchella* yield was the highest in the paddy field, followed by that in the greenhouse, with the lowest yield in the forest and orchard fields (Figure 8A). Linear regression analysis was conducted to investigate the correlation between the alpha diversity of soil bacteria and fungi and *Morchella* yield (Figure 8B; Supplementary Table 2). The results showed that the *Morchella* yield was significantly negatively correlated with soil fungal richness (Chao1 index) and significantly positively related to soil bacterial diversity (Shannon index). Regression analysis was performed to evaluate the correlation between co-occurrence network modules and *Morchella* yield. Surprisingly, no significant correlation was found between any of the network modules and the *Morchella* yield (Supplementary Table 3).

Furthermore, structural equation modeling (SEM) was performed to determine the relationship between the soil microbial community and *Morchella* yield (Figure 8C). Importantly, the results showed that the experimental soil type significantly affected the potassium content of the soil, which could be regulated by changing the soil fungal richness. In addition, the random forest (RF) model was used to predict *Morchella* yield based on the soil microbial community data (Supplementary Table 4). The RF model, which included the bacterial community, had the best prediction ability at the phylum level, but the accuracy was only 37.1%, while the RF model based on the fungal community performed the most accurately at the family level, with a prediction accuracy of 44.9%. In addition, the prediction accuracy of the RF regression analysis obtained by combining the bacterial phylum and fungal family communities for the yield of *Morchella* reached 48.6% (Figure 8D), suggesting that the combination of the bacterial phylum and fungal family communities was more suitable for assessing *Morchella* yield.

## 4 Discussion

### 4.1 The assembly mechanisms of the microbial community in different soil types are not consistent

Regarding ecological processes, there were significant differences in the assembly mechanisms of the bacterial and fungal communities among the different experimental field types for *Morchella* cultivation (Figure 6). Forests are stable ecosystems with strong resilience (Rojas and Stephenson, 2013), and the occurrence of forest disturbances cannot immediately change the diversity and distribution of the bacterial community in local soil. Therefore, *Morchella* cultivation barely impacted soil bacteria, which are shaped by stochastic processes. However, significant differences were observed in the *Morchella* yield collected from the three forest experimental fields (Supplementary Table 1), indicating that there were ecological differences between the forest fields and that these factors led to a deterministic impact on the soil fungal community, thereby directly or indirectly affecting *Morchella* cultivation. Paddies tend to have a wet environment, and soil overlying moisture is an important medium for bacterial transmission, in which ecological dispersal leads to stochastic processes influencing the soil bacterial community. In addition, the paddy field is covered with seedlings, providing a “sheltering effect” for *Morchella* cultivation. A humid and shaded environment is conducive to the growth of *Morchella*, which is consistent with the average yield of *Morchella* in paddy fields being greater than that in other types of experimental fields (Supplementary Table 1).

Notably, the assembly mechanisms of the soil microbial communities in the greenhouse and orchard plots were completely opposite, with deterministic processes shaping the community assembly of bacteria and fungi in the greenhouse, and stochastic processes regulating the assembly of the bacterial and fungal communities in the orchard plot. For greenhouses, increasing temperature leads to an increase in evapotranspiration, reducing the effectiveness and retention time of soil moisture. Furthermore, warming enhances the mineralization of phosphorus and nitrogen, causing nutrient limitations (Wang et al., 2023). These results are consistent with the significantly greater contents of AK, TP, and AAP and lower pH detected in the greenhouse soil than in the other experimental field types. A weakly acidic and enriched nutrient environment in the soil is conducive to bacterial and fungal growth. In addition, there are mostly annual herbaceous plants with shallow roots in greenhouses, while cultivated *Morchella* species are soil saprophytes and have a high mycelium colonization rate, especially at 0–10 cm depths, which enables interactions between mycelia and plant root microorganisms, potentially forming facultative mycorrhizal-like associations with plant roots (Loizides, 2017; Carrasco and Preston, 2020). The construction of a greenhouse results in less external intervention and weaker migration of microorganisms. Thus, the deterministic factors that shape the assembly of bacterial and fungal communities in greenhouses mostly come from soil nutrients and interactions between microorganisms. For orchard, there are mostly perennial shrubs or trees with deep roots, resulting in fewer interactions with mycelia. Previous studies have revealed that different types of compost drive different patterns of microbial communities in the substrate. That is, the growth of *Morchella* does not necessarily depend entirely on the actual soil, and only one or several substrates are needed as carriers to drive the microbial community and provide necessary nutrients for *Morchella* growth (Tan et al., 2021a,b). This may also be one of the important reasons why both bacteria and fungi in orchard are stochastically affected. Furthermore, monoculture of fruit trees caused a decrease in soil fertility and the occurrence of borer disease, further resulting in the lowest yield of *Morchella* (Figure 8A). In addition, soil samples from one field of study are not truly representative, and additional soil samples from different orchard experimental fields will be collected for future research.

It is estimated that 25% of the *Morchella* cultivated area in large-scale outdoor cultivation has been affected by fungal infections (Lv et al., 2022; Shi et al., 2022). From the perspective of ecological assembly, the soil fungal community was shaped mainly by deterministic processes among the different experimental field types (Figure 6). Similarly, previous studies have shown that *Morchella* growth is related mainly to deterministic factors such as temperature, humidity, nutrients, and microorganisms (Liu W. et al., 2017; Longley et al., 2019). This study also indicated that soil potassium, phosphorus, saccharose and starch might be the determining soil factors affecting *Morchella* growth (Figure 1). To this end, changing the deterministic factors in cultivated soil to ensure that *Morchella* or beneficial fungi are abundant as dominant species may constitute a key ecological pathway to optimize *Morchella* cultivation.

### 4.2 Potassium predicts *Morchella* yield by affecting fungal richness

In the present study, the *Morchella* yield was greater in the paddy and greenhouse treatments than in the forest and orchard treatments (Figure 8A). In paddy fields, the cellulose content of the soil was greater than that in other field types (Figure 1), and cellulose decomposes into carbon via a large amount of cellulases in *Morchella* mycelium, providing organic carbon for the surface soil (Xu et al., 2022). Similarly, two unidentified fungi belonging to *Helotiales*, which are major microbial predictors of soil CO_2_ emissions, were significantly enriched in paddies (Newsham et al., 2017). Moreover, members of *Helotiales* are phylogenetically associated with ectomycorrhizae and have a particularly strong ecological link with root endophytes and ericoid mycorrhizal fungi (Tedersoo et al., 2009). *Proteobacteria* and *Actinobacteria* had the highest proportions in the greenhouse and were significantly enriched with o_*Pseudomonadales*, which belong to *Gammaproteobacteria* (Figure 3C). *Pseudomonas* promotes mycelial growth, primordia formation, and high yield (Cho et al., 2003; Kim et al., 2008; Zarenejad et al., 2012). Previous studies have confirmed that *Pseudomonas* are considered the most common bacteria in soil for cultivating *Morchella* and the main biomarker at the primordium stage (Zhang C. et al., 2023). *Pseudomonas* can stimulate sclerotium formation (Hayes et al., 2010), further promote the growth and fruiting of *Morchella* (Pion et al., 2013; Liu Q. et al., 2017; Benucci et al., 2019).

Notably, the SEM results showed that the experimental soil type significantly affected the potassium content of the soil, which could directly or indirectly promote the yield of *Morchella* by inhibiting soil fungal richness (Figure 8C). To our knowledge, this was the first study to predict *Morchella* yield through soil potassium fertilizer and soil fungal community richness, which also emphasized the close relationship between *Morchella* growth and soil microbial ecology. Moreover, *Morchella* growth is related to potassium fertilizer, which is probably linked to “burning morels” phenomenon (Pilz et al., 2004). Fire events produce large amounts of wood ash after fires in spring and summer, and this ash contains a high content of water-soluble potassium and can be used as a fertilizer, further making it possible to grow more *Morchella* in the soil after fires than under conventional cultivation (Li et al., 2017). Several researchers have also simulated the environment after a fire or directly added wood ash when cultivating *Morchella* and found that the growth and development of *Morchella* require a large amount of potassium fertilizer (Zhang C. et al., 2023). Moreover, the soil fungal community was influenced mainly by deterministic processes, and the habitat niche breadth was significantly narrower than that of the bacterial community (Figure 6A), indicating that the fungal community in the cultivated soil of *Morchella* was more sensitive to the bacterial community. Potassium fertilizer changed the ecological niche of the soil fungi, leading to imbalanced competition between the major fungal groups and resulting in changes in *Morchella* yield. The niche width of soil fungi is related to taxonomic richness (Levins, 2020; Zhang C. et al., 2023). Specifically, under suitable cultivation conditions, especially in environments with high potassium fertilizer content, *Morchella* mycelia invade the soil and occupy the soil ecological niche, further becoming the dominant species of the soil microbial community. This was consistent with the greater yield of *Morchella* in the paddy and greenhouse (Figure 8A), where potassium fertilizer is generally applied to increase the aboveground harvest. Most notably, once suitable environmental conditions for pathogenic fungi arise, predominant fungal taxa can inhibit the fructification of *Morchella* (Longley et al., 2019), which may be one of the main potential ecological risks that hinder the *Morchella* agroindustry (Tan et al., 2021a,b).

## 5 Conclusion

Our study demonstrated that soil physicochemical characteristics and microbial diversity and composition exhibited significant difference among the different experimental field types used for *Morchella* cultivation. Although soil physicochemical variables were unlikely to be decisive factors in determining the microbial dynamics in all the experimental field types, the fungal community was more sensitive to the physicochemical variables than the bacterial community. Furthermore, deterministic and stochastic processes together shaped the bacterial community, while the fungal community was governed mainly by deterministic processes among the different experimental field types. These co-occurrence patterns further implied that the soil microbial dynamics are related to fluctuations in the soil physicochemical characteristics. The *Morchella* yield in the paddy and greenhouse treatments was significantly greater than that in the forest and orchard treatments. Notably, the experimental soil type significantly affected the potassium content of the soil, which could directly or indirectly promote *Morchella* yield by inhibiting soil fungal richness. These results provided novel insights into predicting *Morchella* yield through soil potassium fertilizer and soil fungal community richness, which further reveal the relationship between *Morchella* growth and soil microbial ecology.

## Data availability statement

The raw reads obtained from lllumina MiSeq in this study have been deposited into the NCBI Sequence Read Archive (SRA) database under the accession numbers SRP460776.

## Author contributions

YY: Formal analysis, Writing — original draft, Writing — review & editing, Data curation, Methodology, Validation, Visualization. HH: Writing — review & editing, Investigation, Methodology. QW: Writing — review & editing, Project administration, Resources. TX: Writing — review & editing, Formal analysis, Software. YZ: Writing — review & editing, Methodology, Visualization. QC: Writing — review & editing, Data curation, Supervision. HC: Writing — review & editing, Conceptualization, Project administration, Resources, Supervision. JZ: Writing — review & editing, Conceptualization, Funding acquisition, Project administration, Supervision.
