# Supplementary material for: Dynamics of the soil microbial community associated with Morchella cultivation: diversity, assembly mechanism and yield prediction
Source: Front Microbiol. 2024 Feb 15;15:1345231. doi: 10.3389/fmicb.2024.1345231 (PMC10903539; doi:10.3389/fmicb.2024.1345231)
Supplement: Supplementary file 1 [file Data_Sheet_1.docx]

**Supporting Information**





Fig. S1 Boxplots of the alpha diversity of the bacterial (A) and fungal (B) communities among the different soil depths and experimental field types. Error bars represent the standard deviation of the mean (n ≥ 5). Numbers in a rank with different letters indicate a signiﬁcant difference (Tukey's HSD test, *p* < 0.05).





Fig. S2 Principal coordinate analysis (PCoA) of the beta diversity of the bacterial (A) and fungal (B) communities among the different soil depths and experimental field types.





Fig. S3 Effective annotation (A), the coefficient (B) and accumulation (C) curves for the sequencing of bacterial and fungal communities





Fig. S4 The relative abundance of major phyla in the fungal community.

Table S1 Statistics of sequencing data for microbial communities.

| **Sample ID** | **Bacteria** | **Fungi** |
| --- | --- | --- |
| MFD10_1 | 79,488 | 138,108 |
| MFD10_2 | 85,185 | 112,570 |
| MFD10_3 | 77,168 | 92,665 |
| MFD10_4 | 82,292 | 113,496 |
| MFD10_5 | 83,452 | 96,389 |
| MPD10_1 | 86,759 | 125,694 |
| MPD10_2 | 90,928 | 129,001 |
| MPD10_3 | 85,802 | 121,628 |
| MPD10_4 | 93,032 | 109,407 |
| MPD10_5 | 86,446 | 111,137 |
| MPG10_1 | 78,764 | 111,586 |
| MPG10_2 | 78,805 | 105,393 |
| MPG10_3 | 82,087 | 143,263 |
| MPG10_4 | 84,145 | 121,344 |
| MPG10_5 | 80,662 | 145,112 |
| MPS10_1 | 70,603 | 105,055 |
| MPS10_2 | 70,586 | 89,957 |
| MPS10_3 | 77,226 | 137,719 |
| MPS10_4 | 74,157 | 76,095 |
| MPS10_5 | 84,315 | 121,101 |
| MQS10_1 | 66,555 | 66,239 |
| MQS10_2 | 87,208 | 71,056 |
| MQS10_3 | 68,939 | 56,701 |
| MQS10_4 | 81,922 | 77,144 |
| MQS10_5 | 69,171 | 96,659 |
| MQL10_1 | 80,158 | 106,440 |
| MQL10_2 | 83,801 | 104,906 |
| MQL10_3 | 80,891 | 106,009 |
| MQL10_4 | 95,882 | 115,482 |
| MQL10_5 | 70,677 | 83,936 |
| MJL10_1 | 77,629 | 114,653 |
| MJL10_2 | 128,388 | 112,789 |
| MJL10_3 | 93,659 | 92,423 |
| MJL10_4 | 78,178 | 101,040 |
| MJL10_5 | 106,294 | 128,018 |
| MCL10_1 | 81,397 | 133,446 |
| MCL10_2 | 85,224 | 136,166 |
| MCL10_3 | 80,839 | 125,912 |
| MCL10_4 | 83,538 | 145,421 |
| MCL10_5 | 80,442 | 110,747 |
| MFS10_1 | 78,035 | 145,760 |
| MFS10_2 | 78,272 | 140,075 |
| MFS10_3 | 82,125 | 120,901 |
| MFS10_4 | 83,254 | 143,713 |
| MFS10_5 | 88,431 | 132,231 |

Table S2 Correlations between the alpha diversity of microbial communities and the yield of *Morchella*.

| **Type** | **Index** | **R^2^** | ***p-value*** |
| --- | --- | --- | --- |
| Bacteria | Chao1 | -0.039 | 0.853 |
|  | Shannon | 0.617 | **0.015*** |
|  | Pielou_J | 0.030 | 0.193 |
|  | Pd_faith | -0.038 | 0.938 |
| Fungi | Chao1 | 0.251 | **0.005***** |
|  | Shannon | -0.018 | 0.473 |
|  | Pielou_J | -0.037 | 0.777 |
|  | Pd_faith | 0.182 | 0.112 |

Table S3 Correlations between microbial modules and yield of *Morchella*.

| **Module** | **R^2^** | ***p-value*** |
| --- | --- | --- |
| Module 1 | 0.045 | 0.148 |
| Module 2 | -0.040 | 0.916 |
| Module 3 | -0.037 | 0.790 |
| Module 4 | 0.009 | 0.276 |
| Module 5 | -0.037 | 0.777 |
| Module 6 | -0.029 | 0.607 |
| Module 7 | -0.036 | 0.768 |

Table S4 Accuracy of random forest models based on microbial communities to predict the yield of *Morchella*.

| **Type** | **Taxa** | **R^2^** | ***p-value*** |
| --- | --- | --- | --- |
| Bacteria | **Phylum** | **0.371** | **< 0.001** |
|  | Class | 0.262 | 0.006 |
|  | Order | 0.277 | 0.005 |
|  | Family | 0.243 | 0.009 |
|  | Genus | 0.258 | 0.007 |
| Fungi | Phylum | 0.275 | 0.005 |
|  | Class | 0.257 | 0.007 |
|  | Order | 0.401 | < 0.001 |
|  | Family | 0.449 | < 0.001 |
|  | Genus | 0.428 | < 0.001 |
